# Non-Invasive Monitoring of Stromal Biophysics with Targeted Depletion of Hyaluronan in Pancreatic Ductal Adenocarcinoma

**DOI:** 10.3390/cancers11060772

**Published:** 2019-06-04

**Authors:** Ezekiel Maloney, Christopher C. DuFort, Paolo P. Provenzano, Navid Farr, Markus A. Carlson, Ravneet Vohra, Joshua Park, Sunil R. Hingorani, Donghoon Lee

**Affiliations:** 1 Department of Radiology, University of Washington, Seattle, WA 98109, USA; eze@uw.edu (E.M.); vohrar@uw.edu (R.V.); js.park189@gmail.com (J.P.); 2 Clinical Research Division, Fred Hutchinson Cancer Research Center, Seattle, WA 98109, USA; cdufort@fredhutch.org (C.C.D.); pprovenz@umn.edu (P.P.P.); markuscarlson2@gmail.com (M.A.C.); 3 Department of Biomedical Engineering, University of Minnesota, Minneapolis, MN 55455, USA; 4 Department of Bioengineering, University of Washington, Seattle, WA 98195, USA; navid@uw.edu; 5 Public Health Sciences Division, Fred Hutchinson Cancer Research Center, Seattle, WA 98109, USA; 6 Division of Medical Oncology, University of Washington School of Medicine, Seattle, WA 98109, USA

**Keywords:** pancreatic ductal adenocarcinoma, magnetic resonance imaging, hyaluronan, PEGPH20

## Abstract

Pancreatic ductal adenocarcinoma (PDA) is characterized by a pronounced fibroinflammatory stromal reaction consisting of inordinate levels of hyaluronan (HA), collagen, immune cells, and activated fibroblasts that work in concert to generate a robust physical barrier to the perfusion and diffusion of small molecule therapeutics. The targeted depletion of hyaluronan with a PEGylated recombinant human hyaluronidase (PEGPH20) lowers interstitial gel–fluid pressures and re-expands collapsed intratumoral vasculature, improving the delivery of concurrently administered agents. Here we report a non-invasive means of assessing biophysical responses to stromal intervention with quantitative multiparametric magnetic resonance imaging (MRI) at 14 Tesla (T). We found that spin-spin relaxation time T2 values and glycosaminoglycan chemical exchange saturation transfer (GagCEST) values decreased at 24 h, reflecting depletion of intratumoral HA content, and that these parameters recovered at 7 days concurrent with replenishment of intratumoral HA. This was also reflected in an increase in low-b apparent diffusion coefficient (ADC) at 24 h, consistent with improved tumor perfusion that again normalized at 7 days after treatment. Phantom imaging suggests that the GagCEST signal is driven by changes in HA versus other glycosaminoglycans. Thus, multiparametric magnetic resonance imaging (MRI) can be used as a non-invasive tool to assess therapeutic responses to targeted stromal therapy in PDA and likely other stroma-rich solid tumors that have high levels of hyaluronan and collagen.

## 1. Introduction

**Figure 1:**
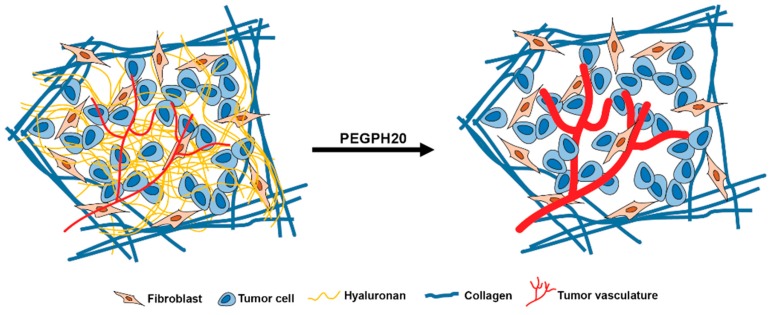
Depletion of hyaluronan with PEGylated recombinant human hyaluronidase (PEGPH20) alters the biophysics of pancreatic ductal adenocarcinoma (PDA). The inordinately high intratumoral gel–fluid pressures in PDA create a physical barrier to diffusion and convection. Targeting hyaluronan in the tumor stroma with PEGPH20 removes this barrier and increases perfusion along with associated changes in quantitative magnetic resonance imaging (MRI) measurements, decreased glycosaminoglycan-spectrum chemical exchange saturation transfer (GagCEST), decreased T2-weighted signal intensity, and an increase in low-b apparent diffusion coefficient (ADC).

Pancreatic ductal adenocarcinoma (PDA) is a highly lethal disease characterized by an unusual capacity for metastatic dissemination and resistance to most forms of chemical and radiotherapies. PDAs are further distinguished by an intense fibroinflammatory infiltrate comprised of several classes of immune cell subsets, fibroblasts in various states of activation, a paucity of vessels, and a complex extracellular matrix that includes high concentrations of hyaluronan (HA), amidst a dense fibrosis. HA is a large, linear, unbranched glycosaminoglycan composed of repeating disaccharide units of N-acetyl glucosamine and D-glucuronic acid that can reach megadalton molecular weight [1,2]. The unusual structure of HA, together with its highly negative charge, contributes to its ability to bind water avidly (up to 15 water molecules per disaccharide unit), resulting in an immobile gel–fluid phase. HA is found in small amounts in virtually all organs of the body, providing turgor and helping to define tissue architecture. In PDA, however, interstitial HA concentrations rival that of the joint space and generate inordinately high swelling pressures that are constrained and counterposed by a tethered collagen fibrillar network that further raises pressures leading to widespread vascular collapse. The resulting hypoperfusion and barriers to diffusion and convection serve as primary mechanisms of resistance to systemically delivered therapies [3,4]. In highly faithful genetically engineered models of autochthonous PDA, administration of PEGylated recombinant human hyaluronidase (PEGPH20) depleted intratumoral HA, lowering interstitial pressures and expanding the vasculature (Figure 1). When combined with a conventional cytotoxic, the resulting increase in intratumoral drug concentrations significantly improved objective response and overall survival [5]. In a phase 1b study in patients with stage IV PDA, PEGPH20 was similarly able to deplete HA in both primary tumors and metastases and significantly increase perfusion; when combined with gemcitabine, patients with the highest intratumoral HA concentrations appeared to benefit the most [6]. In a randomized phase 2 study, the combination of PEGPH20 and gemcitabine + nab-paclitaxel chemotherapy backbone resulted in significantly increased overall survival compared with chemotherapy alone and the largest improvements in progression free survival were again noted in patients with high intratumoral HA [7]. A biomarker-driven global phase 3 trial of PEGPH20 (in combination with gemcitabine and nab-paclitaxel) is currently underway for patients with HA-high stage IV pancreas cancer (HALO-109-301).

The ability of non-invasive imaging strategies to provide functional information on the biophysical properties of solid tumors holds the promise to inform patient selection and provide more rapid assessments of response or resistance. Quantitative magnetic resonance imaging (MRI) can be used to exploit and reveal chemical, structural, and functional properties of healthy and diseased tissues and aid in precision diagnosis and treatment of patient-specific tumor phenotypes [8]. Changes in critical parameters may even predict future benefit or progression prior to the ability to discern such outcomes by purely anatomic measurements. Such modalities will be especially useful as strategies to target key aspects of the tumor microenvironment become increasingly available and important in cancer treatment [9]. In the following, we perform high-magnetic field (14T) multi-parametric quantitative MRI to monitor dynamic changes in the biophysical properties of autochthonous PDAs and normal tissues before and after targeted disruption of stromal HA.

## 2. Results

### 2.1. Apparent Diffusion Coefficient (ADC) Values of PDA

**Figure 2:**
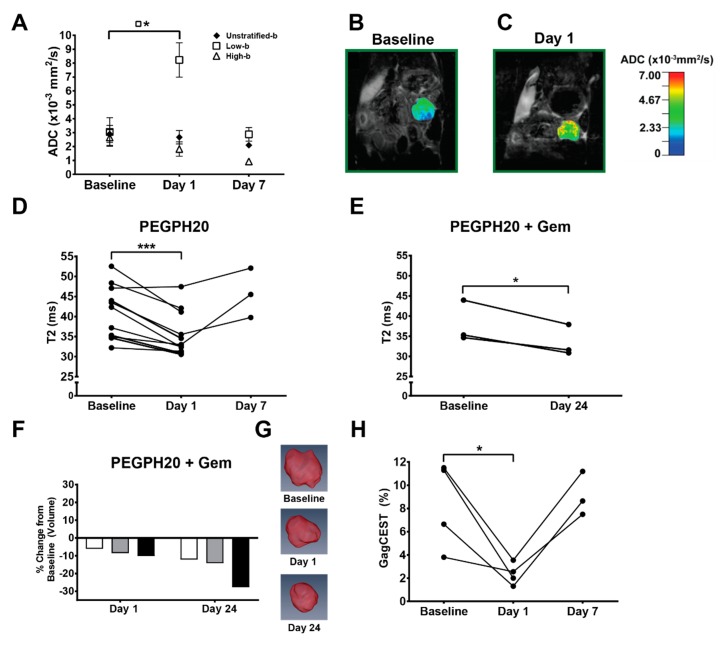
Multiparametric MRI results. (**A**) Unstratified and stratified apparent diffusion coefficient (ADC) values at baseline (*n* = 4), day 1 (*n* = 4), and day 7 (*n* = 3); points = means, error bars = SEM. Low b-values = 7, 47, 81 s/mm^2^. High b-values = 126, 180, 234, 340, 549 s/mm^2^. (**B**,**C**) Representative ADC color-overlay maps on proton density weighted anatomic, coronal plane images from a *KPC* mouse at baseline (**B**) and 24 h (**C**) following PEGPH20 treatment. (**D**) T2 values for animals treated with PEGPH20 at baseline (*n* = 11), day 1 (*n* = 11), and day 7 (*n* = 3). (**E**) T2 values for animals that underwent one full 3-week cycle of PEGPH20 + gemcitabine combination therapy (*n* = 3). (**F**) Tumor volume measurements (*n* = 3) plotted as percent change from baseline for animals that underwent the 3-week PEGPH20 + gemcitabine regimen. (**G**) Three-dimensional volumetric renderings from a representative *KPC* animal treated with PEGPH20 + gemcitabine regimen at baseline, day 1, and day 24. (**H**) Glycosaminoglycan chemical exchange saturation transfer (GagCEST) values at baseline, day 1 and day 7 after PEG monotherapy (*n* = 3). * = *p* <0.05; *** = *p* <0.001.

**Figure 3:**
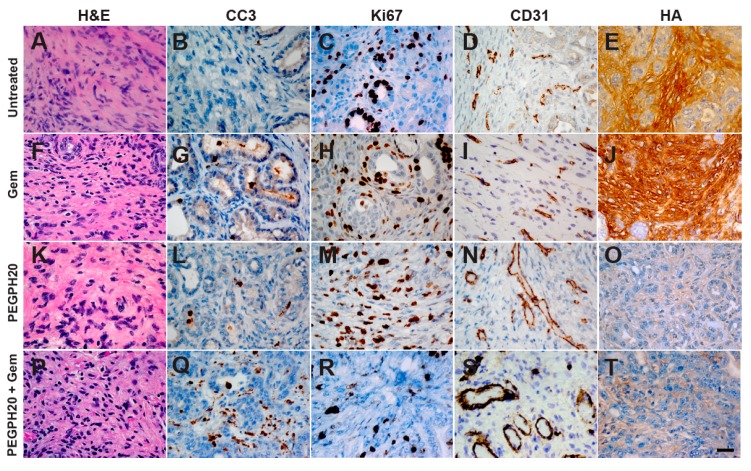
Representative histochemistry of PDA from untreated and treated with gemcitabine (Gem), PEGPH20, or PEGPH20 + gemcitabine *KPC* mice. Untreated *KPC* tumors (**A**–**E**) and gemcitabine administered as a monotherapy (**F**–**J**) show low levels of apoptosis (CC3), increased proliferation (Ki-67), collapsed vasculature (CD31), and high levels of hyaluronan (HA). When treated with PEGPH20, HA is depleted and collapsed tumor vasculature reopens (**K**–**O**) and when administered in combination with gemcitabine after one full treatment cycle there is an increase in apoptosis, decreased proliferation, a re-opening of collapsed vessels, and a depletion of HA (**P**–**T**). Scale bar, 25 μm.

Generation of an ADC map with a bi-exponential model of intravoxel incoherent motion involves terms for both perfusion fraction (“pseudo-perfusion”) and diffusion [10]. At high “b” values (stronger magnetic gradients) the contribution of the perfusion term is minimized, but at low “b” values (e.g., 0–100 s/mm^2^), perfusion effects exponentially dominate ADC values. Twenty-four hours after a single intravenous dose of PEGPH20, tumors showed an elevation in low-b (7, 47, 81 s/mm^2^) mean ADC values (3.05 ± 1.03 baseline versus 8.2 ± 1.2 × 10^−3^ mm^2^/s; *p* = 0.041), consistent with significant degradation of intratumoral HA, expansion of the vasculature and a corresponding increase in perfusion (Figure 2A–C). This effect normalized 7 days post-therapy (2.87 ± 0.49 × 10^−3^ mm^2^/s), as expected from studies of PEGPH20 monotherapy in which intratumoral HA levels recovered within 5–7 days after a single systemic dose of the enzyme [5,11]. Spin-spin relaxation time T2 values also decreased significantly 24 h post-PEGPH20 (41.1 ± 2.0 ms baseline versus 35.6 ± 21.7 ms; *p* = 0.0008) and normalized at 7 days in animals receiving no additional therapy (45.8 ± 3.6 ms) (Figure 2D). T2 values remained decreased, however, in animals that received a full cycle of PEGPH20 + gemcitabine combination therapy (33.5 ± 2.2 × 10^−3^ mm^2^/s; *p* = 0.035) (Figure 2E). Tumor volume also steadily decreased as expected in the latter cohort (91.7 ± 1.3% of baseline at 24 h, *p* = 0.054; 81.9 ± 4.9% of baseline at 24 days, *p* = 0.035) (Figure 2F–G). This is consistent with HA depletion leading to a normalization of tumor vasculature and an increase in perfusion and drug delivery (Figure 3A–T).

### 2.2. GagCEST Imaging of PDA

GagCEST imaging provided quantitative information of intratumoral glycosaminoglycans (“GAGs”, primarily HA) content in response to various interventions. The CEST method overcomes the chemical-concentration limitations of magnetic resonance spectroscopy by capitalizing on normal ^1^H exchange between a chemical branch group of interest and surrounding water molecules. Thus, labile protons on GAGs capable of exchanging with those in water can be used as chemical exchange dependent saturation transfer (CEST) agents, amplifying the signal observed after applying a radiofrequency pulse at a resonant frequency of one of the GAG protons [12]. The continual exchange of saturated protons from the GAG with unsaturated protons from the surrounding water magnifies the saturation effect, conferring a sensitivity approximately two orders of magnitude higher than MR spectroscopy. The inherently low sensitivity of spectroscopy also necessitates long acquisition times, whereas CEST benefits from fast acquisition strategies to achieve superior spatial resolution. The high sensitivity, excellent spatial resolution, and shorter imaging time make CEST an attractive, clinically translatable, chemical quantification MR method [13]. As intratumoral concentrations of HA in PDA, can approach μg/g of tissue, rivaling that of the joint space, GagCEST is a promising method to measure dynamic changes in HA content.

GagCEST values in the autochthonous PDAs also decreased significantly at 24 h post-PEGPH20 (8.31 ± 1.88% versus 2.35 ± 0.47%; *p* = 0.043) and normalized again at 7 days (9.1 ± 1.1%), consistent with depletion and recovery of interstitial HA (Figure 2H). In the animal receiving a full cycle of combined PEGPH20 and gemcitabine, intratumoral levels of HA did not recover resulting in a sustained decrease in the GagCEST value (4.2%) [14]. The spleen served as an internal reference for each animal and generated significantly lower GagCEST quantification values as expected compared to regions of interest in the corresponding invasive PDAs (*p* < 0.01).

### 2.3. GagCEST Imaging of Phantom Tumors

**Figure 4:**
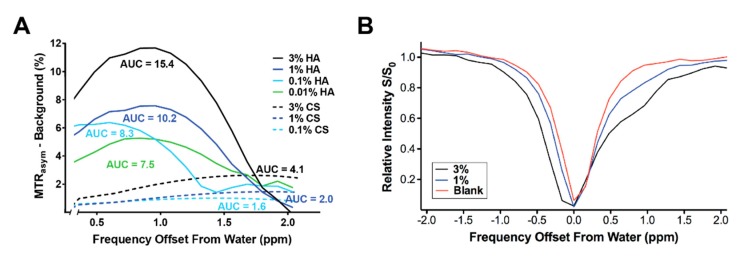
Chondroitin sulfate (CS) and hyaluronic acid (HA) phantom GagCEST characterization at 14T. (**A**,**B**) Magnetization transfer ratio (MTR) asymmetries of CS and HA suspensions minus background. Representative intensity curves from chondroitin sulfate suspensions (1% and 3%) and aqueous blank. Area under the curve (AUC) increased with greater CS and HA content. Note the greater amount of signal generated by HA versus CS, even at 10-fold lower concentration.

We performed GagCEST imaging of CS and HA phantoms to model effects seen in autochthonous tumors and determine their relative contributions to the observed signals. These studies demonstrated a reliable magnetization transfer asymmetry that was greater than that observed with the aqueous “blank” at ≥0.1% concentration for the CS phantom, and at ≥0.01% concentration for the HA phantom between 0 and 2 ppm (Figure 4A,B). Indeed, a larger GagCEST signal was generated by HA than CS at every concentration tested. T1 and MTR values were not significantly different in either treatment cohort at the imaged time points [14].

## 3. Discussion

In contrast with most solid tumors, pancreas cancers decrease perfusion during the course of disease progression [5,15,16]. Moreover, despite their pronounced hypoperfused state, pancreas cancers generate an anti-angiogenic microenvironment. Thus, they possess decreased numbers of vessels that are also less patent than surrounding normal tissues [5,15,16]. These vessels are nevertheless structurally and functionally intact [15]. In other words, pancreas cancer vessels are not “leaky”: they lack the fenestrae and increased interendothelial junctions typical of the abnormal vessels sprouted during the neoangiogenesis observed in many solid tumors. Free fluid pressures in autochthonous PDA are correspondingly low, comparable to those found in normal tissues and lower than those encountered in transplanted xenograft and allograft pancreas cancer model systems [17]. The hypoperfused state in PDA instead arises from vascular collapse of otherwise normal vessels under inordinately high interstitial gel–fluid pressures, themselves the result of the extraordinary swelling forces generated by the unusual properties of hydrated HA in entanglement with a dense collagen fibrillar network [4,17]. A similar organizational architecture was previously described in studies of umbilical cord [18,19]. We have shown here that quantitative MRI can be used to characterize critical properties of intratumoral water content in autochthonous PDA and chronicle fluid shifts between immobile and mobile states, providing crucial dynamic information on the underlying pathophysiology of the disease and the efficacy of stromal targeting therapies.

Targeted depletion of intratumoral HA and mobilization of interstitial gel-fluid resulted in an increase in perfusion-dominant “low-b” ADC values after a single dose of PEGPH20 that returned to baseline in the absence of additional therapy. It has been established that depletion of HA dramatically reduces interstitial gel–fluid pressures, expands the tumor vasculature, and increases perfusion [5,17]. The barriers to perfusion are reconstituted if HA levels are allowed to recover (i.e. in the absence of continued therapy). Increased tumor perfusion and corresponding enhanced drug delivery is consistent with the proposed mechanism of PEGPH20 augmenting chemotherapy regimens in preclinical studies and clinical trials [5,6,15,17,20]. Thus, low-b ADC values may represent a facile means of assessing acute changes in tumor perfusion following PEGPH20 treatment.

The decrease in tumor volume and further decrease in T2 relaxation time seen after one full cycle of combination enzymatic plus cytotoxic chemotherapy were likely due to PEGPH20-augmented delivery of gemcitabine and an associated increase in tumor cell death. Thus, in addition to targeted enzymatic degradation of basal intratumoral HA, HA synthesis in this context is also likely impaired due to both epithelial tumor cell and fibroblast cell death [5].

As described, autochthonous *KPC* PDAs are also known to have significantly elevated interstitial gel-fluid pressures and greater associated hypovascularity compared to other solid tumors and experimental model systems [5,15,16]. We previously identified significantly lower ADC values in autochthonous *KPC* PDAs versus either orthotopic or subcutaneously engrafted tumors in ADC maps generated with b-values of 0, 500, and 1000 s/mm^2^, consistent with decreased Brownian motion and mobility of water [21]. Interestingly, the profound decrease in interstitial gel-fluid pressures and increase in perfusion after PEGPH20 treatment is associated with an increase in free fluid pressures [17]. While overall tumor diffusivity was not significantly changed on high-b ADC values post-PEGPH20 treatment, changes in T2 values clearly reflect this shift in states from immobile to freely mobile fluid within the rigid tumor stroma, with an associated decrease in intratumoral bound water content post-PEGPH20 treatment.

Reliable GagCEST quantification requires adjustments for competing saturation effects including the direct saturation of water (off-target RF saturation) as well as magnetization transfer. Magnetization transfer (MT) imaging can be used to follow the increasing collagen content (fibrosis) associated with disease progression in autochthonous PDA [21]. Thus, together with GagCEST to evaluate glycosaminoglycan content and specifically HA, these modalities can capture the two critical components responsible for the unique mechanobiology of PDA. Similar to CEST, MT utilizes exchange between saturated and unsaturated ^1^H to generate image contrast. However, unlike CEST, MT contrast depends on semi-solid macromolecules with very short T2* times and, hence, involves a very broad spectrum. We have previously shown that acute changes in interstitial pressures after PEGPH20 treatment are associated with a specific loss of intratumoral HA and preserved collagen content, and that at physiological pH, PEGPH20 does not significantly degrade other glycosaminoglycans such as CS A, B, or C in tumor xenografts [17]. Consistent with these prior histochemical quantifications of recovered tissues and *in vitro* enzyme specificity assays, we did not observe differences in MTR before and after acute treatment with PEGPH20. This contrasts with the significant drop observed in GagCEST values and supports the specificity of the CEST measurements for the glycosaminoglycan ^1^H portion of the frequency shift spectrum. Our phantom imaging results further confirm that the signal changes observed in our GagCEST imaging protocol are more greatly influenced by changes in the relative abundance of the HA rather than CS species, which can also be frequently overexpressed in human tumors but exert less potent effects on interstitial pressures [17,20].

## 4. Materials and Methods 

### 4.1. Mouse Strains

All animal studies were approved by the Institutional Animal Care and Use Committee of Fred Hutchinson Cancer Research Center (FHCRC) and the University of Washington (UW). The Office of Laboratory Animal Welfare of the National Institutes of Health has approved Fred Hutchinson Cancer Research Center (A3226-01) and the University of Washington (A3464-01) and these studies complied with the Public Health Service Policy on Humane Care and Use of Laboratory Animals. The *Kras^LSL-G12D/+^;Trp53^LSL-R172H/+^;Cre (KPC)* genetically engineered mouse model has been described previously in detail [22]. These animals conditionally express endogenous mutant *Kras* and point mutant *Trp53* alleles at physiologic levels and faithfully recapitulate the molecular progression and pathophysiology of the human disease from inception to invasion and metastasis. Animals are examined by abdominal palpation and serial high-resolution ultrasound (Vevo 2100; VisualSonics, Inc., Toronto, ON, Canada) beginning at 8–10 weeks of age and are enrolled in preclinical studies when the primary tumor diameter is 3.0–6.0 mm [5,23] (reviewed in [24]). A pegylated formulation of recombinant human hyaluronidase, PH20 (PEGPH20; Halozyme Therapeutics), was administered via bolus tail vein injection at 15 mg/kg.

### 4.2. MRI Protocol

**Figure 5:**
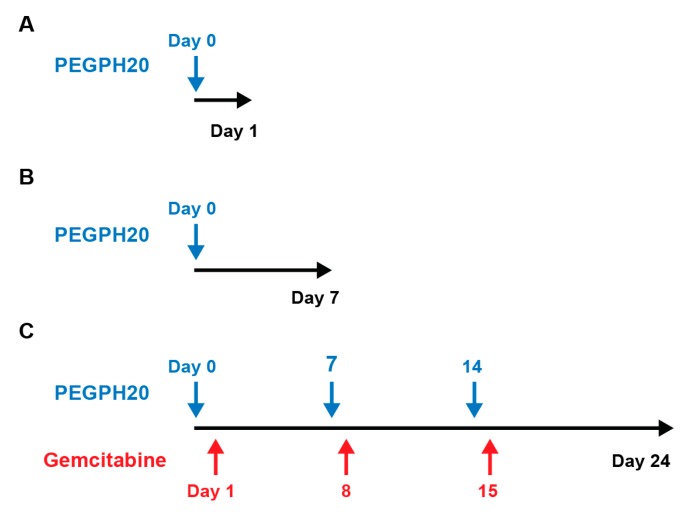
Study timeline. Quantitative magnetic resonance imaging was performed at (**A**) baseline and at 24 h post-PEGPH20 treatment for (*n* = 5) animals, at (**B**) baseline, 24 h, and at day 7, (*n* = 3) and (**C**) after treatment with PEGPH20 and gemcitabine at baseline, 24 h, and at the completion of one full treatment cycle at day 24 (*n* = 3).

MRI was performed on a 14 Tesla (T) Bruker Avance 600 MHz/89 mm vertical bore magnetic resonance (MR) spectrometer (Bruker Corp., Billerica, MA, USA). Mice were anesthetized with isofluorane, with lubrication ointment applied to their eyes, fitted with a respiratory monitoring probe, and then placed inside a birdcage transmit/receive radiofrequency (RF) coil (inner diameter 25 mm). Animals treated with PEGPH20 were imaged at enrollment and also at 24 h (*n* = 11) and 7 days (*n* = 3) after one dose of intravenous PEGPH20 at 24 h (Figure 5A,B). Animals treated with PEGPH20 and gemcitabine were imaged at baseline, 24 h after treatment, and at the completion of one full cycle of treatment (day 24) (*n* = 3) (Figure 5C).

The 14T MRI measurements included T1 weighted (T1W) and T2 weighted (T2W) sequences, as well as quantitative T2 maps. Quantitative MRI methods were applied to generate apparent diffusion coefficient (ADC), glycosaminoglycan chemical exchange saturation transfer (GagCEST), and magnetization transfer ratio (MTR) maps. Whole tumor volumes were reconstructed from T1W tumor anatomy scans using the Amira software platform (Visualization Sciences Group, Burlington, MA, USA). Details of the MRI protocols employed are presented in Table 1 (see also [21,25]). We employed a water saturation shift referencing (WASSR) modification to CEST imaging as described by others for pre-clinical applications at high Tesla magnet strength [26,27,28].

### 4.3. MRI Map Creation and Analysis

Quantitative maps were constructed and analyzed in ImageJ (National Institutes of Health, Bethesda, MD, USA) [29]. T1 and T2 weighted images were used to generate T1 and T2 relaxation maps. GagCEST maps were generated by adapting methods previously validated in human cartilage and glycosaminoglycan phantoms [30,31]. Rather than direct comparison with water, summative saturation sampled at the 0.5, 1.0 and 1.5 ppm frequency shifts was compared to saturation at the opposite frequency shift relative to the central water ^1^H resonance (e.g., signal at +1.0 ppm “*S*_+τ_“ subtracted from −1.0 ppm “*S*_−τ_“, divided by the non-RF-saturated signal “*S*_0_“; formally: (*S*_−τ_ − *S*_+τ_)/*S*_0_) [26,27,28,30,31]. ^1^H MTR maps were generated by subtracting signal intensity of the tissue after the application of the saturation pulse (“*S*_s_“) from baseline signal intensity (“*S*_0_“), relative to baseline: (*S*_0_ − *S*_s_)/*S*_0_. Apparent diffusion coefficient (ADC) maps were generated using a mono-exponential model incorporating the MRI signal intensity with diffusion weighting b (“S_b_”), and the on-diffusion-weighted signal intensity (“S_0_”); formally: S_b_/S_0_ = exp(−b·ADC). A bi-exponential model was used to estimate the intravoxel incoherent motion related parameters of perfusion fraction (or “pseudo-diffusion”) and diffusion with the ImageJ MRI Analysis Calculator plugin [10,32]. Low b values (i.e., <100 s/mm^2^, specifically 7, 47, and 81 s/mm^2^) were used to generate perfusion fraction maps, and higher b values (i.e., >100 s/mm^2^, specifically, 126, 180, 234, 340, 549 s/mm^2^) were used to generate tissue diffusivity maps.

The whole tumor seen on each analyzed slice was included in the region of interest (ROI), and the mean value generated with this ROI was recorded for each analyzed slice. For T1, T2, MTR, and ADC maps, the average value of three separate slices per tumor was used as the single parameter value for the corresponding animal at each time point. GagCEST maps were generated from one slice through each tumor. For statistical testing, we used two-sided paired parametric *t*-tests performed using Prism (v.7; GraphPad Software, San Diego, CA, USA) to compare parameter values obtained at different time points.

### 4.4. Histology and Immunohistochemistry 

Tissues were fixed in 10% neutral buffered formalin, paraffin-embedded, and 5 μm sections were analyzed by histochemistry and immunohistochemistry. Primary antibodies and reagents used were: a biotinylated recombinant HA-binding protein 1:100 (Halozyme Therapeutics HTI-601 [33]), CD31 1:100 (Abcam ab28364), Ki-67 1:150 (Abcam ab16667), and cleaved caspase 3 (CC3) 1:500 (Cell Signaling Technology #9664). Masson’s Trichrome and hematoxylin and eosin (H&E) stains were performed by standard procedures. Concentration matched isotype controls were performed for each primary antibody along with positive and negative normal tissue controls.

### 4.5. Phantom Imaging

Aqueous suspensions of chondroitin sulfate A (CS; Sigma Aldrich, C9819, St. Louis, MO, USA) and sodium hyaluronate (HA; Lifecore Biomedical, HA15M-1, Chaska, MN, USA) were used to construct phantoms for GagCEST characterization at 14T magnet strength. Solutions of varying concentrations (0.01% to 3% by weight) were chosen based on prior *in vitro* and *in vivo* studies of interstitial pressures in genetically engineered models of PDA [17] and GAG content in resected human PDAs [20]. Samples were poured into 5-mm diameter glass tubes (Wilmad-LabGlass, WG-5MM, Vineland, NJ, USA) and immersed in a silicone mold (Dow Corning, Sylgard 184, Torrance, CA, USA).

GagCEST imaging was initially performed using multiple radiofrequency saturation pulse intensities ranging from 1 micro-Tesla (μT) to 5 μT for 1-second pulse duration with nine frequency shifts between –4 parts per million (ppm) and 4 ppm separation from water. For the magnetic transfer ratio asymmetry range of interest (0.5 to 2 ppm), optimal signal-to-noise ratio was achieved with a 4 μT intensity pulse for the CS phantom, and with a 3 μT pulse for the HA phantom, similar to prior validation studies in human cartilage at 7T [31] and in phantoms at 11T [30]. These intensities were subsequently used for further characterization of the CS phantom with 29 frequency shifts between –2.08 and 2.08 ppm, and 34 frequency shifts between −2.04 and 2.04 ppm for the HA phantom.

## 5. Conclusions

In summary, we have shown that a range of non-invasive quantitative MRI modalities can provide detailed mapping of spatial and temporal changes in extracellular matrix components and fluid content during targeted enzymatic depletion of the PDA stroma. T2 maps, low-b ADC maps, GagCEST maps, and tumor volume measurements were combined to comprehensively characterize therapeutic response in a genetically engineered mouse model of PDA. The methods described here can be employed for the non-invasive, rapid evaluation of clinical response to a number of stromal interventions.

## Figures and Tables

**Table 1:** 14T MRI parameter acquisition methods.

Method	Sequence Type	TR/TE (ms)	Comments
T1	RARE	5500, 3000, 1500, 1000, 385.8/9.66	NA = 1; FOV = 30 × 30 mm^2^; rare factor = 2, matrix size = 256 × 128 (reconstructed phase encoding steps = 128; acquisition phase encoding steps = 96); yielding spatial resolution of 0.117 × 0.234 mm/pixel. Approximately 9 min acquisition time.
T2	MSME, fat suppressed	4000/twelve echoes equally spaced from 6.28 to 75.4	NA = 1; FOV = 30 × 30 mm^2^; matrix size = 256 × 128 (reconstructed phase encoding steps = 128; acquisition phase encoding steps = 91); spatial resolution of 0.117 × 0.234 mm/pixel. 10 contiguous slices were acquired with respiration gating to cover the entire abdomen. Approximately 6 min acquisition time.
ADC	EPI	2500/17.7	Echo train length = 16; Pulse duration = 3.0 ms; Diffusion time = 7.46 ms; NA = 1; FOV = 30 × 30 mm^2^; matrix size = 128 × 128; spatial resolution of 0.234 × 0.234 mm/pixel; 8 b values (7, 47, 81, 126, 180, 234, 340, 549) s/mm^2^. Ten contiguous slices were acquired to cover the entire abdomen. Approximately 2 min 40 s acquisition time.
GagCEST	(1) RARE (2) RARE (3) RARE	(1) 2200/7 (2) 5000/7 (3) 5000/7	(1) Center frequency estimate: Continuous-wave block saturation pulse with B1 = 3 μT and duration = 1 s; 25 frequency offsets from −360 Hz to 360Hz with an interval of 0.5 ppm (WASSR approach). FOV = 30 × 30 mm^2^; Matrix size = 128 × 128; Flip angle = 180°; NA = 1. A single, 1 mm slice delineating the tumor was acquired. (2) Frequency shift saturation: Six frequency offsets at ± 0.5, ± 1.0. ± 1.5 ppm were acquired through the same single slice using respiration gating with an off-resonance RF pulse applied for 1 s at a power of 3 μT. Matrix = 128 × 128 (reconstructed phase encoding steps = 128; acquisition phase encoding steps = 96); FOV = 30 × 30 mm^2^; rare factor = 8. (3) Control image: A control image was acquired through the same slice using the same settings as #2, except with saturation offset at 300 ppm.~19 min total acquisition time.
MTR	GRE	625/2	Flip angle = 30°; off-resonance frequency 7000 Hz; saturation pulse block pulse shape = 50 ms width and 10 µT amplitude; FOV = 30 × 30 mm^2^; matrix size = 256 × 256; spatial resolution of 0.117 × 0.117 mm/pixel. Ten contiguous images were acquired to cover the entire abdomen. Approximately 3 min acquisition time.

µT: micro-tesla; ADC: apparent diffusion coefficient; GagCEST: glycosaminoglycan chemical exchange saturation transfer; EPI: echo planar imaging; GRE: gradient echo; mm = millimeters; ms = milliseconds; MSME: multi-slice multi-echo; MTR: magnetization transfer ratio; NA: number of acquisitions; ppm = parts per million; RARE: rapid acquisition with refocused echoes; s = second; SE: spin echo; TE: echo time; TR: repetition time; WASSR: water saturation shift referencing.
